# The incidence of anti-HMGCR immune-mediated necrotizing myopathy: an Australian and UK retrospective multi-site cohort study

**DOI:** 10.1093/rheumatology/keaf238

**Published:** 2025-05-10

**Authors:** Thomas Khoo, Elina Tan, Vidya Limaye, Harsha Gunawardena, Ross Sadler, Janine A Lamb, Xia Lyu, Anna Brusch, Merrilee Needham, Keziah Austin, Aaron Bahadori, Maya H Buch, Maciej Tomaszewski, James B Lilleker, Hector Chinoy

**Affiliations:** Faculty of Health and Medical Sciences, School of Medicine, University of Adelaide, Adelaide, South Australia, Australia; Division of Musculoskeletal and Dermatological Sciences, Faculty of Biology, Medicine and Health, The University of Manchester, Manchester, UK; Rheumatology Unit, Southern Adelaide Local Health Network, Adelaide, South Australia, Australia; Rheumatology Unit, Royal Adelaide Hospital, Adelaide, South Australia, Australia; Department of Clinical Immunology, Sir Charles Gairdner Hospital, Nedlands, Western Australia, Australia; Department of Immunology, PathWest Laboratory Medicine, Perth, Western Australia, Australia; Department of Clinical Immunology, Royal Perth Hospital, Nedlands, Western Australia, Australia; Faculty of Health and Medical Sciences, School of Medicine, University of Adelaide, Adelaide, South Australia, Australia; Rheumatology Unit, Royal Adelaide Hospital, Adelaide, South Australia, Australia; Department of Rheumatology, North Bristol NHS Trust, Bristol, UK; Immunology Laboratory, Churchill Hospital, Oxford University Hospitals NHS Foundation Trust, Oxford, UK; Epidemiology and Public Health Group, School of Health Sciences, University of Manchester, Manchester, UK; Division of Musculoskeletal and Dermatological Sciences, Faculty of Biology, Medicine and Health, The University of Manchester, Manchester, UK; Epidemiology and Public Health Group, School of Health Sciences, University of Manchester, Manchester, UK; Department of Rheumatology, Renji Hospital, Shanghai Jiaotong University School of Medicine, Shanghai, China; Department of Clinical Immunology, Sir Charles Gairdner Hospital, Nedlands, Western Australia, Australia; Department of Immunology, PathWest Laboratory Medicine, Perth, Western Australia, Australia; Neuromuscular Clinic, Perron Institute for Neurological and Translational Science, QEII Medical Centre, Nedlands, Western Australia, Australia; Neuromuscular Clinic, Perron Institute for Neurological and Translational Science, QEII Medical Centre, Nedlands, Western Australia, Australia; Department of Neurology, Fiona Stanley Hospital, Perth, Western Australia, Australia; Institute for Immunology and Infectious Diseases, Murdoch University, Perth, Western Australia, Australia; School of Medicine, University of Notre Dame, Perth, Western Australia, Australia; Department of Rheumatology, North Bristol NHS Trust, Bristol, UK; Fiona Stanley Hospital, Perth, Western Australia, Australia; Division of Musculoskeletal and Dermatological Sciences, Faculty of Biology, Medicine and Health, The University of Manchester, Manchester, UK; NIHR Manchester Biomedical Research Centre, Manchester University Hospitals NHS Foundation Trust, Manchester, UK; Division of Cardiovascular Sciences, Faculty of Medicine, Biology and Health, University of Manchester, Manchester, UK; Manchester Academic Health Science Centre, Manchester University NHS Foundation Trust Manchester, Manchester Royal Infirmary, Manchester, UK; Division of Musculoskeletal and Dermatological Sciences, Faculty of Biology, Medicine and Health, The University of Manchester, Manchester, UK; Manchester Centre for Clinical Neuroscience, Manchester Academic Health Science Centre, Salford Royal Hospital, Northern Care Alliance NHS Foundation Trust, Salford, UK; Division of Musculoskeletal and Dermatological Sciences, Faculty of Biology, Medicine and Health, The University of Manchester, Manchester, UK; NIHR Manchester Biomedical Research Centre, Manchester University Hospitals NHS Foundation Trust, Manchester, UK; Department of Rheumatology, Salford Royal Hospital, Northern Care Alliance NHS Foundation Trust, Manchester Academic Health Science Centre, Salford, UK

**Keywords:** myositis, statins, myopathy

## Abstract

**Objectives:**

Immune-mediated necrotizing myopathy (IMNM) with autoantibodies targeting 3-hydroxy-3-methylglutaryl-CoA reductase (anti-HMGCR) is considered a rare complication of statin therapy. We calculate the incidence of anti-HMGCR IMNM and describe clinical characteristics in four independent cohorts: Manchester (UK), Bristol (UK), Western Australia (WA, Australia) and South Australia (SA, Australia).

**Methods:**

Adults (≥18 years) with anti-HMGCR IMNM (ENMC criteria; 2018–2023) were identified from myositis clinic and laboratory records. Nationwide UK anti-HMGCR testing was performed at Oxford University Hospital Laboratories and state-based WA/SA testing at PathWest Laboratories.

**Results:**

One hundred and nine anti-HMGCR IMNM cases were identified (51% female, median 66 years [IQR 58–72.2]) with median follow-up 2.3 years [IQR 1.5–4.2]. Mean annual incidence was 2.9 cases/million person-years. In statin users, incidence was 20.4 (UK) and 24.1 (WA/SA) cases/million statin-users/year. One hundred and one patients were statin-exposed, mostly atorvastatin (77/101, 76.2%). Median statin duration before diagnosis was 3 years (range: 1 month–23 years). Eight (7.5%) were statin-naïve and, compared with statin-exposed patients, younger (median 46.1 *vs* 67 years, *P* = 0.02), frequently of non-white ethnicity (5/8 *vs* 20/77, *P* = 0.04) and more commonly had dysphagia (4/8 *vs* 14/94, *P* = 0.03). The median peak creatine kinase (CK) was 7020 IU/l (range: 964–39076), and 48/105 (45.7%) received intravenous immunoglobulin. At follow-up, less than half had normal CK (50/105 [47.6%]) or muscle power (48/104 [46.2%]).

**Conclusion:**

For the first time, we have calculated an incidence of anti-HMGCR IMNM using a large, multinational cohort. We highlight the refractory nature of anti-HMGCR IMNM. We also describe the unique phenotype of statin-naïve anti-HMGCR IMNM, and the rare occurrence of self-limiting myopathy.

Rheumatology key messages

- Anti-HMGCR immune-mediated necrotizing myopathy rarely complicates statin use (incidence: 20.4–24.1 cases/million statin-users/year).
- Statin-naïve patients with anti-HMGCR immune-mediated necrotizing myopathy have distinct demographic and clinical characteristics from statin-exposed patients.
- Anti-HMGCR immune-mediated necrotizing myopathy often causes persisting weakness and elevated creatine kinase despite immunomodulatory treatment.

## Introduction

Statins are a class of cholesterol-lowering agents which have become the most prescribed medications globally [1–3]. Through inhibiting the 3-hydroxy-3-methylglutaryl-CoA reductase (HMGCR) enzyme, statins prevent the conversion of HMG-CoA to mevalonate within hepatocytes, thereby decreasing cholesterol synthesis. In response, hepatocytes upregulate low-density lipoprotein cholesterol (LDL-C) receptors which increase uptake of LDL-C, decreasing circulating LDL-C. Statins have revolutionized the primary and secondary prevention of atherosclerotic vascular disease with risk reduction demonstrated over multiple cardiovascular endpoints [4].

Muscle-related adverse effects are the main reasons for patients to stop statins [5]. However, muscle symptoms are much more frequently reported by patients in real-life registries [6] compared with clinical trials where there is generally only a small excess of mostly mild muscle pain attributable to statins [7].

Adverse effects from statins are typically due to myotoxicity causing myalgia, reversible weakness or, occasionally, rhabdomyolysis [8]. Separate from the direct myotoxic effects of statins is immune-mediated necrotizing myopathy (IMNM), part of the group of idiopathic inflammatory myopathies (IIM), involving aberrant immune activation [9].

IMNM is associated with a recognized autoantibody in 88–90% of cases [10]. When occurring with statin exposure, IMNM is characterized by a unique autoantibody targeting the HMGCR enzyme [11, 12]. Notably, anti-HMGCR is highly specific to IMNM and absent in individuals with statin myotoxicity [13].

Anti-HMGCR IMNM is thought to be a rare form of IIM. In a single US centre between 2002 and 2010, 5% of all IIM cases were statin-exposed anti-HMGCR IMNM [12]. In the context of an incidence of 40 cases of IIM/million adults/year in the USA [14], the incidence of anti-HMGCR IMNM was calculated to be two cases/million adults/year [9].

Anti-HMGCR IMNM typically causes severe proximal muscle weakness and marked elevations in creatine kinase (CK) [15]. However, there is growing recognition of rare phenotypic subgroups within anti-HMGCR IMNM. Some patients with anti-HMGCR have a chronic limb-girdle muscular dystrophy-like phenotype [16]. Statin-naïve anti-HMGCR IMNM can occur and seems to be more frequent than statin-exposed anti-HMGCR IMNM in Asian cohorts [17]. In paediatric patients (universally statin-naïve), anti-HMGCR IMNM is infrequent but presents with severe, refractory myopathy [18]. Finally, there are rare cases where anti-HMGCR IMNM is self-limiting [19].

There remain many unanswered questions about anti-HMGCR IMNM, from risk factors to pathogenesis, clinical presentations and treatment strategies [17]. Research into anti-HMGCR IMNM is limited by the rarity of the disease but also by awareness of anti-HMGCR and access to laboratory testing for this autoantibody.

We aimed to assemble the first international, multi-site cohort of patients with anti-HMGCR IMNM, using detailed clinical data to calculate disease incidence and associations.

## Methods

Adult patients (≥18 years) with clinically verified anti-HMGCR IMNM were identified from four regions: two from the UK (Greater Manchester [GM], Bristol/North Somerset/South Gloucestershire—henceforth referred to as Bristol) and two from Australia (Western Australia [WA], South Australia [SA]). Patients with a positive anti-HMGCR result (January 2018–December 2023) were included. Cases were identified through reviewing consecutive, unselected referrals from specialist clinics at each site, cross-referencing with laboratory data of elevated anti-HMGCR antibody levels.

Patients were classified as having anti-HMGCR IMNM according to the 224th ENMC International Workshop criteria [20], namely, a positive anti-HMGCR antibody result, elevated CK and proximal muscle weakness. On review by a clinician with expertise in IIM (H.C., J.B.L., H.G., V.L., E.T., T.K.), patients were excluded if anti-HMGCR was thought to be a false-positive result, i.e. if performed erroneously or as part of a broader panel in the absence of muscle weakness, elevated CK or magnetic resonance imaging evidence of muscle oedema.

In the UK and Ireland, all anti-HMGCR testing is performed at the Oxford University Hospital Immunology Laboratories using chemiluminescence immunoassay (QUANTA flash on BIO-FLASH system, Werfen, Barcelona, Spain) with positive results reported at >20 chemiluminescence units. For WA/SA, anti-HMGCR testing is performed at PathWest Laboratory Medicine Western Australia using ELISA with a positive cut-off of >11 relative units, 3 standard deviations above the mean of a general reference WA population [21, 22]. Anti-HMGCR testing using ELISA/chemiluminescence immunoassay has high specificity and has previously been validated with immunoprecipitation [23, 24].

Demographic data, statin-related factors (specific statin, dose/duration of use), comorbidities, laboratory results (peak CK, other autoantibody specificities [tested using line immune-assay], and histological diagnosis [20, 25]) and treatment regimens were documented. Core set measures of disease activity as defined by the International Myositis Assessment and Clinical Trials group were not reliably documented for all patients, and consequently, modified outcomes were used: whether CK levels normalized (<200 IU/l for females and <320 IU/l for males, noting that the catchment populations of all sites were of predominantly white ethnicity) and whether power normalized at latest clinical review.

Notable subgroups were identified: statin-naïve patients, and patients who appeared to have self-limiting myopathy that resolved to normal muscle power and CK without immunosuppression.

Incidence was calculated using the latest census data (2021) for England (Office for National Statistics, www.ons.gov.uk/census) and Australia (Australian Bureau of Statistics, www.abs.gov.au/census). The populations of GM, Bristol, WA and SA remained relatively stable from 2018 to 2023, with essentially distinct populations and minimal migration between the sites. Census data are published for people aged 20 years and older which, for the purposes of calculating incidence, is a close approximation of the adult population.

In the UK, general practice prescribing data reported that ∼10% of the total population were prescribed a statin in 2019 [1]. In Australia, prescription medications are accessed via the government supported Pharmaceuticals Benefits Scheme (PBS). The number of statin users was determined from a PBS data query (Supplementary Data S1, available at *Rheumatology* online) from 2019 to 2023, noting that data for 2018 are no longer accessible. The incidence of anti-HMGCR IMNM in statin users was calculated using population statin usage data.

Descriptive statistics were applied. Fisher’s exact test was used to compare categorical variables. For continuous variables, the Mann–Whitney *U*-test was used to produce a *z*-score, referring to the sum of the ranks within the group of interest, and resultant *P*-value. *P*-values <0.05 were considered statistically significant. Stata version 18 (StataCorp LLC, College Station, TX, USA) was used for statistical analysis.

Finally, national level data were obtained from the Oxford University Hospital Immunology Laboratories for the entire UK (England, Wales, Scotland, Northern Ireland [2018–2022]) and state-based data from PathWest Laboratory Medicine for WA/SA (2018–2023). The population incidence of positive testing for anti-HMGCR was then calculated using census data.

This research was conducted according to the 1964 Declaration of Helsinki and all later amendments. The retrospective data collection for this study was approved by the Central Adelaide Local Health Network Human Research Ethics Committee (ref. 18934), the Sir Charles Gairdner Hospital and Osborne Park Health Care Group Human Research Ethics Committee (RGS3141) and was registered as a collaborative Quality Improvement project between Bristol and the Northern Care Alliance NHS Foundation Trusts (24HIP10).

## Results

There were 109 clinically verified cases of anti-HMGCR IMNM diagnosed from 2018 to 2023 (GM, 34; Bristol, 17; WA, 35; SA, 23) with a median of 2.3 years follow-up (interquartile range [IQR] 1.5–4.2). Fifty-one (46.8%) were female and the median age was 66 years (IQR 58–72.2). The combined cohort was predominantly white (87/109, 79.8%) although WA had a higher proportion of patients of non-White ethnicity (12/35, 34.3%), eight of whom were Aboriginal and/or Torres Strait Islander peoples. Table 1 summarizes the demographics, statin exposure, comorbidities and treatments.

**Table 1.** Demographic, statin-related, comorbidity, laboratory and clinical outcome data of patients with anti-HMGCR immune-mediated necrotizing myopathy

	Greater Manchester	Bristol, North Somerset and South Gloucestershire	Western Australia	South Australia	Combined
Adult population^a^, *n*	2 143 400	758 900	2 000 979	1 379 223	6 282 502
Anti-HMGCR positive cases, *n*	34	17	35	23	109
Demographics					
Female, *n* (%)	20 (58.8)	9 (52.9)	14 (40)	8 (34.8)	51 (46.8)
Age^b^, median (IQR), years	65.8 (60.4–72.6)	61 (56–69)	64 (56–71)	70.3 (65.8–73.6)	66 (58–72.2)
Non-White ethnicity, *n*	6 (African, Pakistani [3], Indian, Chinese)	1 (Chinese)	12 (Macedonian, Malaysian, Asian, African, Aboriginal [8])	3 (Papuan, Aboriginal [2])	22
Statin, *n*					
Atorvastatin	21	15	25	16	77
Rosuvastatin	1	1	3	4	9
Simvastatin	3	0	1	0	4
Unknown statin	5	1	3	2	11
Statin duration prior to anti-HMGCR testing, median (range), years	4 (1–15)	3 (0.25–23)	3 (0.5–20)	2 (0.1–10)	3 (0.1–23)
Statin-naïve, *n*	4	0	3	1	8
Diabetes, *n* (%)	13/31 (41.9)	9/17 (52.9)	23/35 (65.7)	11/23 (47.8)	56/106 (52.8)
Malignancy, *n* (%)	2 (5.9)	3 (17.6)	4 (11.4)	8 (34.8)	17 (15.6)
Cancer-associated myositis^c^, *n* (%)	0 (0)	0 (0)	4 (11.4)	4 (17.4)	8 (7.3)
Peak CK, median (range), IU/l	5500 (1000–39 076)	8276 (3083–20 000)	7095 (2900–35 000)	7000 (964–17 109)	7020 (964–39 076)
Dysphagia, *n* (%)	6/31 (19.4)	0/17 (0)	9/35 (25.7)	3/21 (14.3)	18/103 (17.5)
Other autoantibodies, *n* (%)	4 (11.8) (PL7, weak; PMScl75, weak; RNP; chromatin and Sm-RNP)	1 (7.1) (MDA5, weak)	1 (2.9) (TIF1γ)	3 (13.1) (SSA [2], TIF1γ)	9 (8.3)
Proportion of muscle biopsies^d^ showing IMNM, *n* (%)	11/17 (64.7)	6/6 (100)	10/14 (71.4)	19/21 (90.5)	46/58 (78)
Treatment exposure					
IVIg, *n* (%)	5/31 (16.1)	6/17 (35.3)	24/35 (68.6)	13/23 (59.1)	48/105 (45.7)
Rituximab, *n* (%)	3/31 (9.7)	7/17 (41.2)	5/35 (14.3)	3/23 (13)	18/105 (17.1)
Outcomes					
CK normalized with treatment, *n* (%)	10/30 (33.3)	8/17 (47.1)	24/35 (68.6)	8/23 (34.8)	50/105 (47.6)
Power normalized with treatment, *n* (%)	8/30 (26.7)	9/16 (56.3)	19/35 (54.3)	12/23 (52.2)	48/104 (46.2)
Ongoing prednisolone use >12 months after diagnosis, *n* (%)	8/14 (57.1)	5/13 (38.5)	12/26 (46.2)	5/14 (35.7)	30/67 (44.8)

a Population from 2021 census data. Adult population defined as people aged 20 years and above to align with census age categories.

b At time of anti-HMGCR testing.

c Defined as malignancy occurring within three years prior to, or following, diagnosis of inflammatory myopathy.

d Where muscle biopsy was performed. HMGCR: 3-hydroxy-3-methylglutaryl-CoA reductase; IMNM: immune-mediated necrotizing myopathy; IVIg: intravenous immunoglobulin.

### Incidence

Annual incident cases of anti-HMGCR IMNM are shown in Table 2. Although Bristol and SA reported generally stable annual cases, GM had more cases in 2022 (10 cases) compared with any other year (3–6 cases/year), and WA more cases in 2021 (15 cases) compared with any other year (2–7 cases/year).

**Table 2:** Annual and average incidences of anti-HMGCR immune-mediated necrotizing myopathy in adults (per million person-years)

Year	Greater Manchester		Bristol		Western Australia		South Australia		**Combined incidence** ^a^
	Cases	**Incidence** ^a^	Cases	**Incidence** ^a^	Cases	**Incidence** ^a^	Cases	**Incidence** ^a^	
2018	6	2.8	4	5.3	7	3.5	3	2.2	3.2
2019	3	1.4	2	2.6	2	1.0	6	4.4	2.1
2020	4	1.9	3	4.0	2	1.0	2	1.5	1.8
2021	6	2.8	4	5.3	15	7.5	2	1.5	4.3
2022	10	4.7	1	1.3	2	1.0	5	3.6	2.9
2023	5	2.3	3	4.0	7	3.5	5	3.6	3.2
2018–2023		2.6		3.7		2.9		2.8	2.9

a Incidence (per million person-years) calculated using 2021 census data of people 20 years and older, accessible from the Office for National Statistics, UK (www.ons.gov.uk/census) and Australian Bureau of Statistics, Australia (www.abs.gov.au/census). HMGCR: 3-hydroxy-3-methylglutaryl-CoA reductase.

Incidence ranged from 2.6 (GM) to 3.7 (Bristol) cases/million person-years. The combined incidence from all four sites was 2.9 cases/million person-years and was essentially equivalent between England (2.92 cases/million person-years) and Australia (2.86 cases/million person-years).

In the statin-user populations, there was an incidence of 20.4 cases in GM/Bristol (1 in 49 114 statin-users/year) and 24.1 in WA/SA (1 in 41 531 statin-users/year) of anti-HMGCR IMNM/million statin-users/year (Supplementary Data S2, available at *Rheumatology* online).

From population testing data, nationwide tests for anti-HMGCR in the UK increased from 980 (2018) to 2918 (2022) and in WA/SA, from 299 (2018) to 552 (2023); 7.7% of total samples in the UK, and 4.4% in WA/SA tested positive for anti-HMGCR, yielding an incidence of 2.1 positive anti-HMGCR tests/million/year (UK) and 4.8 positive anti-HMGCR tests/million/year (WA/SA).

### Statin exposure

The most common statin exposure was atorvastatin (77/101, 76.2%), although rosuvastatin (9/97, 9.3%) and simvastatin (4/97, 4.1%) were also represented (Table 1). The duration of statin use prior to detection of anti-HMGCR antibodies varied from 1 month to 23 years, with median time of 3 years. The median dose of atorvastatin was 40 mg daily (range: 10–80 mg) and for rosuvastatin, 10 mg daily (range: 5–20 mg).

Eight patients (7.3%) were statin-naïve (Table 3). When compared with statin-exposed patients, the statin-naïve subgroup was younger (median age 46 [IQR 38.5–63.8] *vs* 67 [IQR 60–72.3] years, *z* = 2.3, *P* = 0.02) and more likely to be of non-White ethnicity (5/8 *vs* 20/77, *P* = 0.04) comprising three patients of Asian and two of African ethnicity. There was no difference between statin-naïve and statin-exposed groups in peak CK level (median 7055 *vs* 7020 IU/l, *z* = −1.1, *P* = 0.3) or anti-HMGCR titre (UK: median 167.5 *vs* 150 CU, *z* = 0.4, *P* = 0.7; Australia: median 35.7 *vs* 31.5 RU, *z* = 0.8, *P* = 0.4). Dysphagia was more commonly experienced by statin-naïve patients (4/8 *vs* 14/94, *P* = 0.03). Diagnostic re-labelling of myopathy subtype after anti-HMGCR testing was more common in the statin-naïve group (5/8 [prior diagnoses: polymyositis (2), dermatomyositis, limb-girdle muscular dystrophy, tropical infection] *vs* 16/101 [prior diagnoses: other IIM subtype (11), myofibrillar myopathy (2), rhabdomyolysis (2), autoimmune hepatitis (1)], *P* = 0.007). Muscle biopsies were performed more commonly in the statin-naïve group (7/8 *vs* 51/101) although not reaching statistical significance (*P* = 0.06).

**Table 3.** Statin-naïve anti-HMGCR immune-mediated necrotizing myopathy patients

ID	Age, years, gender	Ethnicity	Peak CK, IU/l	Dysphagia	Biopsy performed	Prior diagnosis	Medications at onset of myopathy
GM1	19, F	Asian	39 076	N	Y	DM	None
GM13	28, F	African	7109	N	Y	PM	None
GM27	61, F	White	4646	N	Y	LGMD	Levetiracetam 750 mg twice daily
GM63	49, F	Asian	4686	Y	Y	—	None
WA5	42, M	White	7000	Y	Y	—	None
WA11	80, M	Asian	15 300	Y	N	DM	Triptorelin 22.5 mg 6 monthly; mirabegron 50 mg daily; metoprolol 12.5 mg twice daily; apixaban 5 mg twice daily; multivitamin daily
WA15	43, M	African	40 600	Y	Y	Tropical infection	Perindopril 10 mg daily
SA4	73, F	White	6558	N	Y	—	Imatinib 400 mg daily; pantoprazole 20 mg daily; perindopril 8 mg daily

CK: creatine kinase; DM: dermatomyositis; HMGCR: 3-hydroxy-3-methylglutaryl-CoA reductase; LGMD: limb-girdle muscular dystrophy; PM: polymyositis.

### Self-limiting disease

A particularly rare subgroup was identified comprising five patients with seemingly self-limiting disease (Table 4). The reasons for avoidance of immunosuppression included being assigned an alternative diagnosis to IIM, minimal muscle weakness, or exhibiting clinical improvement at the time of clinic review. The self-limiting subgroup had statin exposures ranging from 2 to 6 years prior to diagnosis with anti-HMGCR IMNM. When comparing individuals with self-limiting and persistent myopathy, there was no difference in age (median 67.2 *vs* 65.5 years, *P* = 0.6), peak CK (median 5734 *vs* 7064 IU/l, *P* = 0.7), anti-HMGCR titre (UK: insufficient data; Australia: median 24.9 *vs* 31.5 RU, *z* = 0.6, *P* = 0.5) or comorbid type 2 diabetes (4/5 *vs* 52/101, *P* = 0.4). The median time to normalization of CK in the self-limiting myopathy group was 334 days (IQR 322–335). One patient had persistently raised CK for 4 years prior to the detection of anti-HMGCR, after which statins were stopped, and CK normalized after 398 days, without immunosuppression.

**Table 4.** Cases of self-limiting anti-HMGCR immune-mediated necrotizing myopathy

Patient ID	Age, years, gender	Statin exposure, duration	Peak CK, IU/l	Time to normalization, days
GM8	67, M	Atorvastatin, 6 years	2413	335
GM10	77, M	Simvastatin, 6 years	17 000	322
WA29	58, F	Atorvastatin, duration unknown	4680	334
WA37	75, M	Rosuvastatin, 5 years	5734	1907^a^
SA13	61, M	Atorvastatin, 2 years	8711	168

a WA37 had an elevated CK detected four years prior to anti-HMGCR testing, which normalized 398 days after anti-HMGCR positive result. CK: creatine kinase; HMGCR: 3-hydroxy-3-methylglutaryl-CoA reductase.

### Comorbidities and cancer

Comorbid type 2 diabetes affected 56/106 (52.8%) of the cohort (Table 1), although less prevalent in statin-naïve than statin-exposed patients (1/7 *vs* 55/99, *P* = 0.05). Statins were mainly prescribed for primary prevention of vascular disease (62/96, 64.6%).

The malignancy rate varied between individual sites, ranging from 5.9% (GM) to 34.8% (SA), with 17 patients (15.6%) having a diagnosed malignancy. Eight (7.3%) satisfied the commonly used cancer-associated myositis (CAM) definition, namely, malignancy within 3 years of IIM diagnosis [26]. Both haematological (chronic myeloid leukaemia) and solid organ (gallbladder, endometrial, renal cell, skin, bowel, hepatocellular, prostate and bladder) malignancies occurred.

Our reported CAM rate (7.3%) is higher than governmental estimates of the 5-year prevalence (2016–2020) of cancer in England (3.5% [27]) and the point-prevalence (2022) of cancer in Australia (4.1% [28]) for people ≥45 years old.

### Laboratory investigations

All patients in this cohort had elevated CK during the disease course, with median peak CK 7020 IU/l (range: 964–39 076). Myositis-specific (anti-Mi2, NXP2, SAE1, MDA5, TIF1γ, Jo1, PL7, PL12, EJ, OJ, KS, Zo, Ha, SRP) and myositis-associated (anti-Ro52, U1RNP, Ku, PmScl, cN1A) antibodies in addition to anti-HMGCR were rare, only occurring in nine (8.3%) patients. Two patients had anti-TIF1γ antibodies (typically associated with dermatomyositis and CAM), neither of whom had a rash, although one had locally advanced bladder/prostate cancer, resected 1 month prior to anti-HMGCR testing.

Anti-HMGCR titre was available for 83/109 patients (76.1%) with the remainder being reported as positive without an exact titre. Anti-HMGCR titres, where available, were strongly positive: in the UK, median 150.9 CU (IQR 105.5–200, positive reported at >20 CU) and in Australia, median 33.1 RU (IQR 25.5–35.7, positive reported at >11 RU).

Muscle biopsy was performed in 58 (53.2%) patients with 46 (78%) biopsies having a histological diagnosis of IMNM. Of the remaining 12, eight showed inflammatory myopathy without features of a specific histological subtype, two demonstrated non-specific myopathic changes, and one was suggestive of inclusion body myositis (IBM).

### Treatment and outcomes

Of the patients treated with immunosuppression, most received prednisolone (88/91, 96.7%). Two patients with mild weakness were successfully treated with methotrexate monotherapy and another patient rapidly responded to intravenous immunoglobulin (IVIg) without needing steroid therapy.

Methotrexate was the most common DMARD (66/91, 72.5%). Almost half of this cohort were treated with IVIg, with Australian sites using IVIg more frequently than centres in the UK (WA, 68.6%; SA, 59.1% *vs* GM, 16.1%; Bristol, 35.3%). Rituximab was uncommonly used except in Bristol (WA, 14.3%; SA, 13%; GM, 9.7% *vs* Bristol, 41.2%).

Less than half showed evidence of disease improvement at the time of latest clinical review, with CK normalizing in 50/105 (47.6%) and power in 48/104 (46.2%) of patients. Peak CK level was not associated with whether patients achieved normal CK (*P* = 0.8) or normal power (*P* = 0.6). Reviewing patients with >12 months of documented follow-up, almost half (30/67, 44.8%) remained on prednisolone therapy. Of the patients who were no longer on prednisolone at 12 months, 15/34 (41.2%) were on a single DMARD, most commonly methotrexate (11/15, 73.3%). The remaining 19 patients were all being treated with additional IVIg and/or rituximab.

## Discussion

We report detailed clinical characteristics and the most precise estimate to date of disease incidence in patients with anti-HMGCR IMNM. As the first multinational cohort study in anti-HMGCR IMNM, from four specialist neuromuscular sites in two separate countries, we have assembled the largest multicentre anti-HMGCR cohort to date. We also report anti-HMGCR testing data on a nationwide (UK) and state-based (WA/SA) scale, made possible by the unique situation of single, centralized testing laboratories.

Our calculated average incidence of anti-HMGCR IMNM in the current combined cohort, 2.9 cases/million/year, is higher than that reported from the Johns Hopkins myositis cohort (from 2002–2010, 2 cases/million/year) [9] and in England (in 2019, 1.94/million adults/year) [29]. The incidence in New Zealand, 4 cases/million/year (October 2018–September 2021), is higher than our incidence, additionally reporting a five times higher incidence in Polynesian compared with European populations [30].

Our calculated incidence reiterates the rarity of anti-HMGCR IMNM. The variation in case numbers from year to year, noted in the GM and WA cohorts, highlights the importance of considering incidence over an extended period rather than point incidence, especially for rare conditions.

When reviewing UK nationwide data from the central testing laboratory in Oxford, the incidence of a positive anti-HMGCR result (2.1/million/year) was similar to the calculated incidence of clinically verified anti-HMGCR IMNM from GM/Bristol (2.92/million/year). In contrast, WA/SA positive anti-HMGCR testing (4.8/million/year) was higher than the calculated incidence (2.86/million/year). This disparity may reflect the influence of geography on case ascertainment. In Australia, despite large geographical areas, specialist healthcare with access to anti-HMGCR testing is highly concentrated in the capital cities.

Our clinical data confirm the general understanding that anti-HMGCR IMNM is characterized by proximal muscle weakness and elevated CK levels. Dysphagia was uncommon, and absent in the Bristol cohort, but more frequent in statin-naïve patients. However, there was limited information on how consistently patients were asked about swallowing symptoms, assessed by speech therapists, or tested with fluoroscopic-based swallow studies. Previous cohorts have reported a significant disparity between subjective and objective dysphagia in patients with anti-HMGCR IMNM [31].

Muscle biopsy was performed in approximately half of the cohort, IMNM being the most common histological diagnosis. Despite one case having histological features of IBM, no clinical features consistent with IBM have developed over 4 years of subsequent follow-up. Notably, the latest ENMC criteria do not require a muscle biopsy to be performed to diagnose anti-HMGCR IMNM [20].

Diabetes was present in more than half of our cohort but is confounded by indication since guidelines encourage a lower threshold to consider statin prescription in patients with diabetes [32]. Diabetes was a less commonly encountered comorbidity in statin-naïve than statin-exposed individuals.

Previously, some cohorts have reported an association of anti-HMGCR with elevated malignancy risk [33, 34] while others have not [15, 29, 31, 35]. Small sample size in anti-HMGCR IMNM cohort studies complicates the reliable calculation of cancer risk, resulting in wide confidence intervals. The older age of our cohort and bias of enhanced surveillance after a diagnosis of anti-HMGCR IMNM influence cancer prevalence. Although our reported rate of CAM is higher than the governmentally reported prevalence of cancer in the ≥45 years age group in both countries, our rate is comparable to an American cohort with anti-HMGCR IMNM which reported rates of cancer (6/104, 6%) matching the general population [19].

Most patients in our cohort were statin-exposed, with atorvastatin being the most implicated drug, consistent with previous cohorts [29, 36–38]. In Australia, the two most-prescribed medications, atorvastatin and rosuvastatin, are used relatively evenly [39] while in England, atorvastatin is the most prescribed medication by a large margin [40]. Notably, some patients in our cohort used rosuvastatin, suggesting that anti-HMGCR IMNM is not limited to patients on lipophilic statins.

Statin-naïve individuals with anti-HMGCR IMNM were younger than the statin-exposed group as reported in other cohorts [22, 34, 41]. A higher frequency of statin-naïve anti-HMGCR IMNM is observed in Asian cohorts [34, 42–45], but it is not known whether this can be extrapolated to other non-White ethnicities. Although theorized that naturally occurring dietary statins may drive the development of anti-HMGCR in statin-naïve individuals [17], a detailed dietary history was not consistently collected in our cohort. Urine testing for statins is possible [46]; a future study performing this test in statin-naïve patients with anti-HMGCR IMNM could objectively assess for any prescribed, illicit or dietary statin intake.

In our cohort, anti-HMGCR IMNM was generally refractory with almost half of individuals with >12 months follow-up still requiring prednisolone at latest clinical review. Use of specialized therapies was high, with almost half receiving IVIg, a treatment usually reserved for patients with severe and/or refractory disease. Variation between GM/Bristol and WA/SA in rituximab and IVIg use likely reflects differences in policies regarding access.

Unlike anti-SRP IMNM, which almost universally involves marked CK elevation and profound weakness [47], the heterogeneity of anti-HMGCR IMNM calls for personalized approaches to treatment. Research is needed to identify biological and genetic predictors of disease severity and treatment response.

Our results prompt consideration of novel hypotheses regarding the pathogenesis of anti-HMGCR IMNM. The variability in duration and dose of statin exposure prior to disease onset suggests other environmental, drug-related or patient-centric factors contributing to autoimmunity. The higher prevalence of non-White ethnicity in the statin-naïve sub-group raises the possibility of ethnic variation in genetic risk or environmental exposures. The unique description of self-limiting myopathy with anti-HMGCR positivity implies novel immunological mechanisms distinct from the traditional paradigm of self-perpetuating autoimmunity and inflammation in the setting of the anti-HMGCR autoantibody.

A key strength of our study is the collaborative approach to assembling a sizeable cohort of patients with a rare condition. This, together with the cross-checking of detailed clinical data, has enabled a rigorous enquiry into the incidence and associations of anti-HMGCR IMNM. Assessing absolute numbers of anti-HMGCR IMNM cases in discrete geographical areas with accompanying census data obviates sampling error, thereby improving the accuracy of incidence calculations. Additionally, a single testing laboratory for the UK, and for WA/SA, allows the determination of anti-HMGCR incidence on a population scale.

There are some salient limitations. Although we are confident of robust case ascertainment for our cohort through clinical and serological verification, it is impossible to confirm complete case ascertainment. GM, Bristol, SA and WA all have specialized centres with neuromuscular expertise and established referral pathways for sub-specialist input at the request of both primary and secondary healthcare providers. The use of separate data sources for case identification (myositis clinic records and serological data) improves the accuracy of capture. However, as demonstrated by the increasing number of tests performed for anti-HMGCR in the UK and WA/SA annually, changing awareness and access to testing affects accurate case identification. In particular, the statin-naïve sub-group may be underestimated given a lack of awareness about, and testing for, anti-HMGCR in statin-naïve individuals with IMNM.

Additionally, calculations of age and statin duration were determined according to the date of anti-HMGCR testing which may not be accurate for the onset of myopathy. Although the use of a multi-national, multi-site approach improves the sample size and statistical power of our results, our cohort was predominantly White and therefore still lacks generalizability to other ethnicities. Finally, the different methods of testing between the UK and Australia mean that it is difficult to directly compare quantitative anti-HMGCR results.

## Conclusions

Anti-HMGCR IMNM has an incidence of 2.9/million person-years in the adult population, and 20.4–24.1/million person-years in the statin-user population. Although rare, anti-HMGCR IMNM caused persistent weakness and reliance on glucocorticoids in approximately half of patients in this study. Therefore, maintaining vigilance and undertaking investigations in patients with a suggestive clinical phenotype is important to enable early diagnosis and treatment.

Unique subgroups of anti-HMGCR IMNM include statin-naïve patients and individuals with self-limiting disease who do not require immunosuppression to recover. Although progress has been made in formulating treatment and cancer-screening guidelines, there remains a significant research need to refine, improve and personalise management of patients with anti-HMGCR IMNM. Ultimately, further international collaborations are needed to bring further light to patients developing anti-HMGCR IMNM, overshadowed by the importance of statins and obscured by rarity, but with great potential to grow our understanding of the complex interactions between statins, muscle and the immune system with focused translational research.

## Supplementary Material

keaf238_Supplementary_Data

## Data Availability

The data that support the findings of this study are available from the corresponding author, upon reasonable request.
