# Supplementary material for: The incidence of anti-HMGCR immune-mediated necrotizing myopathy: an Australian and UK retrospective multi-site cohort study
Source: Rheumatology (Oxford). 2025 May 10;64(9):4995–5003. doi: 10.1093/rheumatology/keaf238 (PMC12407235; doi:10.1093/rheumatology/keaf238)
Supplement: keaf238_Supplementary_Data [file keaf238_supplementary_data.zip › keaf238_Supplementary_Data/rhe-25-0220-File006.docx]

**Supplementary Material File 1**

**Services Australia Data Query – Statin Prescriptions in Western Australia and South Australia**

**Request summary (run date 4^th^ October 2024)**

Request: Number of unique patients prescribed a statin in each calendar year (2019-2023) from Western Australia and South Australia

Patient location: State (Western Australia [WA] and South Australia [SA]) on the 31^st^ of December of each calendar year

Year of Supply: 2019, 2020, 2021, 2022, 2023

Pharmaceutical Benefits Scheme (PBS) codes: 13479K, 9053L, 13384K, 9054M, 13536K, 9055N, 9056P, 13596N, 9049G, 13567C, 9050H, 13415C, 9051J, 13597P, 9052K, 13495G, 8213G, 9230T, 13529C, 8214H, 9231W, 13468W, 8215J, 9232X, 13374X, 8521L, 9233Y, 10376D, 13538M, 10392Y, 13539N, 10393B, 13622Y, 10377E, 13416D, 10208G, 13569E, 10201X, 13480L, 10207F, 13537L, 10204C, 13629H, 13586C, 2484B, 2628H, 13588E, 2574L, 2609H, 13589F, 2594M, 2636R, 13406N, 2590H, 2606E, 13385L, 9483D, 13442L, 9484E, 13535J, 8881K, 13595M, 8882L, 13528B, 2011W, 9242K, 13373W, 2012X, 9243L, 13471B, 8173E, 9244M, 13471B, 8173E, 9244M, 13471B, 8173E, 9244M, 13559P, 2013Y, 9241J, 13498K, 8313M, 9245N, 13496H, 2833D, 9237E,13497J, 2834E, 9238F, 13432Y, 8197K, 9239G, 13527Y, 8829Q, 9240H, 13558N, 28673Q and 9236D

Caveats: PBS only (excluding Repatriation Pharmaceutical Benefits Scheme [RPBS] and Under Co payment data)

**Data output:**

| **Patient_State** | **Year_of_Supply** | **Number of unique patients prescribed a statin (n)** |
| --- | --- | --- |
| SA | 2019 | 149,432 |
| SA | 2020 | 173,450 |
| SA | 2021 | 178,021 |
| SA | 2022 | 184,297 |
| SA | 2023 | 190,679 |
|  |  |  |
| WA | 2019 | 161,126 |
| WA | 2020 | 195,674 |
| WA | 2021 | 203,417 |
| WA | 2022 | 212,502 |
| WA | 2023 | 220,312 |
