# Supplementary material for: The incidence of anti-HMGCR immune-mediated necrotizing myopathy: an Australian and UK retrospective multi-site cohort study
Source: Rheumatology (Oxford). 2025 May 10;64(9):4995–5003. doi: 10.1093/rheumatology/keaf238 (PMC12407235; doi:10.1093/rheumatology/keaf238)
Supplement: keaf238_Supplementary_Data [file keaf238_supplementary_data.zip › keaf238_Supplementary_Data/rhe-25-0220-File007.docx]

**Supplementary Material File 2**

**Calculation of incidence of anti-HMGCR immune-mediated necrotising myopathy (IMNM) per statin-user population**

**England:**

Population of Greater Manchester and Bristol* = 2,867,800 + 979,500 = 3,847,300 people

10% of patients in England using statins [1] = 384,730 statin users

From the present study, 30 (Greater Manchester) + 17 (Bristol, North Somerset and South Gloucestershire) = 47 patients with statin-exposed anti-HMGCR+ IMNM between 2018 and 2023 🡪 average number of cases per year = 47/6 = 7.8 cases/year

Incidence of anti-HMGCR immune-mediated necrotising myopathy per statin-users per year = 7.8 cases/384,730 statin users x 1,000,000 = **20.4 cases of anti-HMGCR IMNM per million statin users per year, or 1 in 49,114 statin-users per year**

**Australia:**

Population of Western Australia and South Australia* = 2,660,026 + 1,781,516 = 4,441,542 people

Average number of people in Western Australia and South Australia prescribed a statin between 2019 and 2023^#^ = 373,782

From the present study, 32 (Western Australia) + 22 (South Australia) = 54 patients with statin-exposed anti-HMGCR between 2018 and 2023 🡪 average number of cases per year = 54/6 = 9 cases

Incidence of anti-HMGCR+ IMNM per statin-users per year = 9 cases/373,782 statin users x 1,000,000 = **24.1 cases of anti-HMGCR IMNM per million statin users per year, or 1 in 41,531 statin-users per year**

*Population data obtained from publicly available 2021 census data (UK – Office for National Statistics, [www.ons.gov.uk/census](http://www.ons.gov.uk/census); Australia – Australian Bureau of Statistics, [www.abs.gov.au/census](http://www.abs.gov.au/census)).

^#^Data on statin users obtained from an Australian Government Services Australia External Information Request (see Supplementary Material File 1 – Services Australia Data Query).

**References:**

1 Curtis HJ, Walker AJ, MacKenna B, Croker R, Goldacre B. Prescription of suboptimal statin treatment regimens: a retrospective cohort study of trends and variation in English primary care. Br J Gen Pract 2020;70(697):e525-e33.
